# Experiences of Weight-Loss Surgery in People With Serious Mental Illness: A Qualitative Study

**DOI:** 10.3389/fpsyt.2020.00419

**Published:** 2020-05-12

**Authors:** Susanna Every-Palmer, Sarah E. Romans, Richard Stubbs, Anneka Tomlinson, Sophie Gandhi, Mark Huthwaite

**Affiliations:** ^1^Department of Psychological Medicine, University of Otago, Wellington, New Zealand; ^2^Wakefield Obesity Surgery, Wakefield Hospital, Wellington, New Zealand; ^3^Department of Psychiatry, University of Oxford, Oxford, United Kingdom

**Keywords:** mental illness, psychosis, obesity, bariatric surgery, qualitative research, discrimination

## Abstract

**Background:**

Bariatric surgery is seldom accessed by people with serious mental illness, despite high rates of obesity in this population. It is sometimes assumed that patients with complex psychiatric histories will have poor post-surgical weight loss or exacerbation of psychiatric symptoms, although this is unsubstantiated.

**Objectives:**

A qualitative descriptive study to explore personal experiences and the impact of bariatric surgery on physical and mental well-being and life-quality in individuals with serious mental illness.

**Methods:**

Nine adults with a history of bariatric surgery and concurrent severe depressive disorder, bipolar disorder, or schizoaffective disorder were interviewed about their experiences of bariatric surgery and its outcomes using semi-structured interview schedules. Data were transcribed and inductive thematic analysis undertaken.

**Results:**

Five broad themes emerged: (1) surgery was highly effective for weight loss, and resulted in subjective improvements in physical health, quality of life, and mental health described as *being able to live a life*; (2) recovering from surgery was a *tough road*, notably in the post-operative period where negative sequelae often anteceded benefits; (3) post-operative support was important, but sometimes insufficient, including from families, mental health services, and surgical teams; (4) most considered surgery life-changing, recommending it to others with mental illness and obesity, two had different experiences; (5) participants considered it discriminatory that people with mental illness were not referred or declined weight loss surgery.

**Conclusions:**

Participants benefited from bariatric surgery and felt it should be offered to others with mental illness, but with additional care and support.

## Introduction

People living with serious mental illness (SMI), including psychotic disorders and bipolar disorder, have a high prevalence of obesity—nearly twice the rates of the general population (1, 2). There is convincing evidence that SMI in general and certain antipsychotic medications in particular, are associated with high risks of metabolic syndrome—a constellation of cardiovascular risk factors including abdominal obesity, dyslipidemia, hypertension, and hyperglycemia (3–5). This contributes to the elevated mortality from cardiovascular diseases (1), with the presence of SMI itself being a risk for adverse cardiac outcomes over and above that predicted by other established risk factors (6). Obesity and its correlates are major contributors to the dramatic reduction in life expectancy in this group (7, 8). This means evidence-based interventions for weight loss need to be carefully considered, researched, and promoted for those with SMI. Unfortunately, this important research base is limited.

Lifestyle and pharmacological interventions have been shown to result in modest weight loss in people with SMI—on average 3 kg (9–12), but data are inconsistent (13, 14) with little long-term cardiometabolic follow-up.

In the general population, bariatric surgery has superior weight loss outcomes compared with other interventions for severe obesity, resulting in substantial improvements in comorbid conditions (15, 16) and quality of life (15, 17). While less severe conditions such as anxiety, mild to moderate depression, and binge eating disorder are common amongst bariatric surgery patients with a corresponding body of research (18–20), the eligibility of people with more severe psychiatric conditions is controversial (21–23) and research in this group is surprisingly scant.

It is often assumed people with complex psychiatric histories will have poor post-operative weight loss and higher complication rates, which may result in their not being referred or being declined surgery (24, 25). In a survey of mental health professionals conducting bariatric surgery eligibility evaluations, 91.2% identified “psychiatric issues” as “clear contraindications” to weight loss surgery (25).

In fact, what research there is suggests that those with SMI have comparable weight loss and complication rates to controls although unsurprisingly people with SMI may require more postoperative support (26–31). In a systematic review of eight eligible studies of bariatric surgery in people with schizophrenia or bipolar disorder, Kouidrat and colleagues reported favorable weight loss outcomes (32). However most included studies focused solely on physical endpoints like weight loss without assessing changes in mental state (32). Research investigating the subjective peri-operative experiences of patients with SMI is distinctly absent. This study aims to examine the personal experiences of bariatric surgery on physical and mental health, and on quality of life in patients with a history of SMI.

## Materials and Methods

### Participants

We included participants who had undergone bariatric surgery within 15 years and had active diagnoses of bipolar disorder, psychotic disorder, or severe depressive disorder at the time of the surgery, identified by ICD or DSM diagnosis (in the medical records) or by medication use (therapeutic doses of mood stabilizer and/or antipsychotic medication), with subsequent confirmation on interview. All had been assessed as having stable symptoms at the time of surgery, competent to consent, and capable of complying with postoperative care.

This study received ethical approval from the University of Otago Human Ethics Committee (Health), ref H17/116.

Consent to participate in the study and for publication of data was obtained from all participants.

### Recruitment and the Interviewing Process

Patients were recruited through convenience sampling from patient records or snowballing techniques. They were contacted by researchers *via* postal invitations containing study information, or *via* telephone, and invited to participate. After obtaining written informed consent, SG conducted semi-structured interviews face-to-face or telephonically, if preferred by participants. Interviews were conducted in and by a university department of psychological medicine.

Interviews were audiotaped and transcribed verbatim within 4 days of being conducted. Data were entered into NVivo qualitative data analysis software (NVivo, version 10, QSR International).

### Analysis

Data were analyzed using thematic analysis (33). Thematic analysis is an established method for identifying, reporting, and analyzing patterns within data, with minimal organization and description of the data in rich detail. Patterns are identified iteratively through a careful process of data familiarization, data coding, theme development, and review (33).

We used an inductive, realist approach to data analysis, in which we reported the experiences, meanings, and the reality of participants (34). The inductive approach involves coding the data without trying to fit it into a pre-existing coding frame or the researcher’s analytic preconceptions. We selected this thematic approach *a priori*, as those of us designing the interview (SEP, MH, SR) and conducting the analysis (SEP, MH, SR, and AT) were theoretically agnostic about bariatric surgery. We have backgrounds in psychiatry with limited experience of obesity surgery and wanted to be guided by our participants’ experiences. We selected a strongly data-driven approach as the most appropriate method to explore personal experiences and the impact of bariatric surgery on physical and mental well-being and quality of life.

Two researchers (SEP and MH) independently read and coded transcripts; codes were examined and iteratively condensed into groups capturing similar themes, each with a number of subthemes. These themes and sub-themes were then reviewed by members of the wider team (SR and AT). To achieve saturation of themes, we moved back and forth between data collection and analysis, ensuring the fit between data and the conceptual work of analysis and interpretation. Interviews completed early in the study informed subsequent data collection, and analysis.

## Results

### Participants

Sixteen potentially eligible patients were identified from patient databases (*n* = 13) and snowballing techniques (*n* = 3). Nine responded affirmatively and met eligibility criteria. Demographic characteristics are summarized in **Table 1**. On average, 7 years had passed since surgery. Primary psychiatric diagnoses included bipolar disorder (*n* = 5), schizoaffective disorder (*n* = 1), and severe depressive disorder (*n* = 3), with psychosis in five. All were prescribed psychotropic medication: antipsychotics (*n* = 5); mood stabilizers (*n* = 7); and antidepressants (*n* = 6).

**Table 1:** Demographic and clinical features of participants.

Participant number, gender	Age range^I^	Diagnosis (at the time of surgery)	Psychiatric medication at time of surgery	Time since surgery (years)	Type of surgery	BMI at surgery (kg/m^2^)	BMI present time (kg/m^2^)	Greatest % total weight loss (any time post surgery)	% total weight loss (current time)	Change in BMI (kg/m^2^)	% Excess weight loss
1F*	30–40	Schizo-affective disorder	Olanzapine Sodium valproate	10	Laparoscopic band (reversed)	38.2* (est.)	44.1* (est.)	26.9	-15.4	−5.9* (est.)	−44.6%* (est.)
2M	60–70	Bipolar disorder	Moclobemide Lamotrigine	10	Gastric bypass surgery	42.0	30.7	31.5	26.8	11.2	66.2
3F	60–70	Bipolar disorder with psychotic features	Olanzapine Lithium Venlafaxine	9	Fobi pouch	50.2	31.3	38.5	37.7	18.9	75.1
4F	20–30	Severe depression	Venlafaxine Mirtazapine Clonazepam	2	Fobi pouch	44.3	31.9	40.6	28	12.4	64.2
5F	50–60	Severe depression with psychotic features	Quetiapine Clomipramine	4	Fobi pouch	53.4	36.5	32.2	32.2	16.9	59.5
6F	50–60	Severe depression	Sodium valproate Amitriptyline	8	Fobi pouch	70.6	39.0	50.6	44.8	31.7	69.4
7F	50–60	Bipolar disorder with psychotic features	Chlorpromazine Lithium Nortriptyline	12	Fobi pouch	49.2	45.8	29.1	7.0	3.46	14.3
8F	40–50	Bipolar disorder with psychotic features	Quetiapine Lithium	6	Laparascopic band	43.9	30.4	46.9	31	13.5	71.4
9M	40–50	Bipolar disorder	Lithium	1	Fobi pouch	38.8	29.0	32.3	24	9.8	71.0

BMI, body mass index; est, estimated; F, female; kg, kilogram; m, meter; M, Male.

^I^Exact ages not given to protect anonymity.

*Accurate BMI records not available for Participant 1. Weight available, height estimated at 165 cm, the average height of a New Zealand woman in order to provide BMI estimates.

Participants described significant mental illnesses that started early in adult life and pre-dated surgery.

“I would have been 19–20 [when diagnosed] and it was very severe and I was [responding to command hallucinations] … floridly psychotic and I stopped eating, I stopped showering, I stopped doing everything [8].”

Participants reported significant functional impairment that impacted on activities of daily living, relationships, and ability to work.

“I was quite ill mentally. And I wasn’t leaving the house. Slept most of the day and most of the night. And was in a really bad place [4].”

Prior to surgery, seven of the nine participants had been hospitalized for mental health crises, with two in long-term inpatient care. One participant had received long term care from forensic mental health services. Five had histories of suicide or self-harm attempts.

### Weight Prior to Surgery

Prior to surgery participants had an average body mass index (BMI) of 48 kg/m^2^ (**Table 1**). Psychotropic medication was viewed as a major causal factor for weight gain.

“I’d spent a year in [hospital] and they put me on quetiapine and quetiapine is a shocking weight gainer. And I put on … I got to 130 kg, I mean I was heavy [8].”

“I was on olanzapine. I’d always had really low weight. I weighed 60 or 50 kg and then it went up to 104…and it kept going up [1].”

All described functional impairment and obesity-related physical health issues, including diabetes, obstructive sleep apnea, and impaired mobility. Participants had tried multiple weight loss strategies, which they found ineffectual.

### Surgery

Participants thought carefully about surgery before proceeding. All self-funded surgery, in most cases with financial support from relatives, after having been declined or believing they were ineligible for publicly funded surgery.

“My parents paid for it … they felt it was worth it to help me because I was in such a bad place [4].”

Two participants traveled several hundred kilometers to providers that accepted patients with stable SMI.

“I got turned down from the [public hospital] in town here [because of] … my psychiatric problems … I was a bit annoyed really. Because I’m just a human like the next woman that wants helps with their obesity … [3].”

The type of bariatric surgery included laparoscopic gastric bypass surgery (*n* = 1), laparoscopic adjustable gastric banding (*n* = 2), and open Fobi pouch gastric bypass (*n* = 6). One participant subsequently had a gastric band removal.

### Themes

We identified five major themes as follows: [1] Surgery was highly effective across multiple domains extending beyond weight loss; [2] the post-operative period was challenging; [3] support was important and needed to be multidisciplinary; [4] most people thought surgery was life-changing and recommended it to others with SMI and obesity; [5] it was considered discriminatory that people with SMI were not referred or actively discouraged from weight loss surgery. These themes are explored below, and supporting quotes appear in **Table 2**.

**Table 2:** The five key themes and examples of supporting quotes.

**Theme 1: “It worked!”**	

**Subtheme: I lost weight and that was great**	*“[Before surgery I couldn’t] wipe my bum … cut my toenails … pick anything up off the floor … put my knickers on easily … [Now] I’m active … I can bend over and put on my shoes. I can do all the things I couldn’t do before … It’s just like I’m a different person [5].”* *“My husband was actually pushing me around in wheel chair [before surgery] and now I’m up and walking and doing everything everyone else is … Look I’m 67, 68 this year and I can ‘out-walk’ most of the old girls as I call them [3].”* *“There’s things like being able to play with my child, comfort getting down on the ground, getting up, ease of walking up hills, better sex life [9].”* *“I can walk upstairs now; my knees don’t hurt. I can pick up kids from the ground … I can move around freely. I don’t feel like my arms are hitting the doorways when I walk through [4].”* *“He [the surgeon] performed a miracle on me and he gave me back my life. I didn’t have a life back then … I could barely get anywhere … I remember going to the lake here years ago with a couple of my children … and I sat down on the grass and it suddenly dawned on me—I didn’t know how I was going to get up off the ground. It makes me feel really emotional talking about it now … I was so fat and so bloody tired [6].”* *“The desire to eat reduced enormously [2].”* *“I’ve always been an overeater and a compulsive binger before the surgery and now I find that I can eat compulsively if I need to, but I don’t put on weight. I don’t much, but if I do, there’s not the consequences there used to be [5].”* *“I still look in shop windows now to make sure I’m still thin [6].”*
**Subtheme: My health improved and I’m going to live longer**	*“My sleep apnea improved; my asthma improved … My peak flow was better. Yeah, so it was an all-round improvement [8].”* *“I don’t have type 2 diabetes which is what I had before. Within days, it was gone [7].”* *“I don’t have a blood pressure problem anymore, I’m no longer pre-diabetic … It’s just like I’m a different person. I don’t get breathless, I don’t have sleep apnoea. My lungs are fine now. … I don’t get asthma now, so I haven’t needed to take Ventolin [5].”* *“When I arrived in Wellington [for surgery], I had injections for diabetes but by the time I left the following Wednesday, I didn’t have to have them anymore [6].”* *“Basically, all my readings [HbA1c, cholesterol, BP] are now normal … [I have] increased my expected life expectancy, probably 10–15 years in my opinion [9].”* *“I believe if I wasn’t dead, I’d be bloody close to it, if I hadn’t had that operation [6].”*
**Subtheme: My quality of life is better**	*“I’ve gone from zero to ten in my quality of life [5]”*. *“I’ve got a job, which I would have never of got being as big as I was. Everything really got better. I could look after the house better. I could look after my family better … I was happy to go out [6].”* *“I could shop in normal shops. Didn’t have to go to special ‘big girl shops’ and that was amazing [4].”* *I remember on Christmas day … it was the first time I was wearing a brand-new dress, brand new me, and everyone was really supportive, and I feel that was the turning point with my mind and body. The connection with my weight and depression [4].”* *“My relationships with my family and friends improved [8].”* *“I have found the love of my life, we’re getting married next year … I feel comfortable touching my partner and … that would have never happened before [4].”* *“I’m not sitting around doing nothing, not able to do anything. I’ve got a life. So, my behavior has improved a lot. And I’ve made friends, I’ve made several friends [5].”*
**Subtheme: My mental health has improved**	“*I feel like when you’re looking at me, you can go on my personality a bit, rather than immediately looking at me and seeing me as obese [4].”* *“My mood … has been a lot better since the surgery … I’m still on meds but it’s had a huge impact on my mental health … My self-esteem is really good. I want to live rather than wanting to die. Huge changes [5].”* *“I’ve got a better frame of mind [3].”* “*It made it [my mental state] better in the long term … I’ve moved out of home. When I was at my worst, I thought I’d never leave, thought my Mum would be my carer for the rest of my life. It’s better, it’s just better [4].”*
**Theme 2: Don’t get me wrong—it was a tough road**	

	*“I was on antipsychotics and antidepressants. Quite big doses … and then with the general anesthetic I was delusional, hallucinating after the surgery … I still needed my psych meds and I couldn’t drink water or swallow, so he had to leave a drain into my old stomach through which we syringed the meds … To start with I was a bit screwed up. A bit angry and found it hard, but I’m so glad I did it [5].”* *“The surgery itself—it hurt like heck! It was very, very tough in the first few weeks [4].”* *“It’s a bit of a tough road after surgery … but so worth it [5].”* *“I was losing weight immediately and I could see it every day just getting better. I looked good. I thought, ‘Am I a failure?’ because my mind wasn’t getting better [4].”* *I suppose I was a bit naïve really. I thought my biggest problem was that I was overweight and thought—I really thought—that I wouldn’t be depressed [if I lost weight] and I’d be deliriously happy, but it didn’t quite work like that [6].”* *“I used to vomit all the time [after surgery]. My friends would be embarrassed, I was embarrassed, it was unpleasant. People would talk about it [8].”* *“I’m still really careful today especially if I go out because the last thing you want to do is be hanging over the toilet at somebody’s house [6].”* *“So, I’m regularly drinking more alcohol, probably not that often to the point of being intoxicated, but I think there might be grounds for that connection of transfer of addiction from what was quite strong food addiction to drinking extra alcohol [9].”* *“I’m an addictive personality. I did have a period where I drank a lot … But anyway, I’ve stopped drinking completely now. I haven’t drunk for six months and I’m not going to [5].”*
**Theme 3: Peri-operative support is really important**	

	*“I was a bit befuddled. I had to take my husband to explain it a bit more … not that they didn’t explain it properly, I didn’t take it in properly [3].”* *“I had support. I had my mum and I had a counselor at the time. If I didn’t have people to talk it through, then I would have regretted it. I’d probably be worse—I don’t know what would have happened [4].”* *“I don’t think you realize how much support you need afterwards. Just someone to talk to about what’s going on [4].”* *“…After the op, I really thought there wasn’t a lot back up. It would have been good to meet a few people and have this support group because you just have to figure it out yourself [7].”*
**Theme 4: Value of surgery**	

**Subtheme: It was all worth it—do it!**	*“So, my life turned around … My mum died four and a half years ago, and I was so pleased that mum was able to see me slim before she died. Because I know—I was 50 years old when mum died—just over, and I know my mum used to worry her guts out about me. Mums worry their guts out about kids forever. It was just the best really. It was the best decision we ever made [6].”* *“…I would recommend it to anybody…. you still have issues; you still have to look after controlling your eating and all the rest of it, but the benefits have been quite significant [2].”* *“I would say, make sure you have the support. Do it. Do it, it’s amazing [4].”* *I can’t eat a lot of the food my family eats, but I would not ever change what I did. Not ever … I just would not swap for anything, not even for money. That’s how much it has helped me in my life [6].”* *“I’m really glad I did the full thing, that [my surgeon] did with the whole open surgery … I think some of the others are a bit transitory and don’t last [5].”*
**Subtheme: It wasn’t worth it—don’t do it**	*“I get my medication daily because at one stage, this was after the op, I was in tremendous pain and I was eating every pill … I was just taking them like lollies [candy] and it wasn’t making the pain any better [7].”* *“My olanzapine and Epilim … was not getting into my stomach … So, three days after the surgery … I suddenly went really manic. And I thought what the hell was wrong with me? … But my weight did go down to 70 kgs. I was pleased, but just unwell just all the time. So, I got it taken out … and once the band came out the medication started to work, pretty much straight away … [If people with SMI have surgery] the drugs won’t work and the person will be even sicker. But if [if they don’t have surgery] they gain too much weight then they’ll get diabetes and die anyway … you’ve got to weigh it up. [1].”*
**Theme 5: The health system is discriminatory**	

	*“I think that it’s just wrong to group people and say, ‘you’ve had mental illness, not going to do surgery…’ Because for me, I wouldn’t be here if I didn’t have the surgery. It makes me a bit sad that if I didn’t have my parents and I was to go public, they would just say ‘no’ and I would be still be stuck where I was. And that was living hell. I would really like it to change. It saved my life; it can save someone else’s life [4].”* *“I think it should be the opposite. I think that the people with mental illnesses and bad self-esteem because of their weight should be the first on the list because it has such a huge drastic change in your, um, your being able to live a life, being able to live an active, enjoyable life and self-esteem. [5]*. *“I think it’s disgusting. It’s not a choice we make to feel this way and it’s not fair to judge and to say, ‘you are depressed so we can’t deal with you’ [6].”* *“It’s a mistake and they should reassess … it would be hugely beneficial to New Zealand hospitals to make this surgery available publicly to much higher number of people [9].”*

#### Theme 1: It worked!

Seven participants (78%) reported the operation was successful, improving their health and lives in the medium to long term (“being able to live a life”). This was the predominant theme, shared by the majority, with strong convergence in accounts. Most reported profound improvements across multiple areas (classified as subthemes)—weight loss, broader physical health, quality of life and mental health. Two people had differing experiences, which are discussed in Theme 4.

##### Subtheme 1(a): I lost weight and that was great

All nine participants lost weight after surgery. The average *greatest* percentage weight loss was 36.25%. The average *current* percentage weight loss was 27.2%. Eight participants weighed significantly less now than prior to surgery. The participant who had a gastric band removal had regained weight. Weight loss had profound effects, allowing people to achieve things they considered important, but could not formerly manage:

“[Before surgery I couldn’t] wipe my bum … cut my toenails … pick anything up off the floor … put my knickers on easily … [Now] I’m active … I can bend over and put on my shoes. I can do all the things I couldn’t do before … It’s just like I’m a different person [5].”

“He [the surgeon] performed a miracle on me and he gave me back my life. I didn’t have a life back then … I could barely get anywhere … It makes me feel really emotional talking about it now … I was so fat and so bloody tired [6].”

##### Subtheme 1(b): My health improved and I’m going to live longer

All but two participants reported net positive improvements in physical health (e.g. *“hugely beneficial,” “an all-round improvement”*). Eight participants reported improvements in obesity-associated morbidities such as sleep apnea, asthma, pre-diabetes, or diabetes. Most described requiring fewer medications or lower doses, particularly antihypertensive and diabetic control medications.

“When I arrived [for surgery], I had injections for diabetes, but by the time I left the following Wednesday, I didn’t have to have them anymore [6].”

People believed their health gains would extend their lifespan:

“Basically all my readings [HbA1c, cholesterol, BP] are now normal … [I have] increased my expected life expectancy, probably 10–15 years in my opinion [9].”

“I believe if I wasn’t dead, I’d be bloody close to it, if I hadn’t had that operation [6].”

##### Subtheme 1(c): My quality of life is better

Seven participants said their quality of life had improved, describing improvement in vocational, functional, and social domains.

*“I’ve gone from zero to ten in my quality of life [5]”*.

“I’ve got a job, which I would have never got being as big as I was. Everything really got better. I could look after the house better. I could look after my family better [6].”

Improvements in self-confidence, self-esteem, and self-image, led to positive changes in social life and overall quality of life. Many felt personal and family relationships improved.

“I’m not sitting around doing nothing, not able to do anything. I’ve got a life. So, my behavior has improved a lot. And I’ve made friends, I’ve made several friends [5].”

##### Subtheme 1(d): My mental health improved

Most participants, with one notable exception, reported improvements in their long-term mental health following surgery, relating to indirect effects, such as greater self-confidence and social connections. Physical changes gave people a greater sense of identity, and the hope they could be viewed on their own merits, rather than stigmatized as overweight psychiatric patients.

*“I feel like when you’re looking at me, you can go on my personality a bit, rather than immediately looking at me and seeing me as obese [4]”*.

The sense of regeneration was reflected in the recurring phrase “different person,” with positive effects cited on mental health and wellbeing.

*“It made it [my mental state] better in the long term … I’ve moved out of home. When I was at my worst, I thought I’d never leave, thought my Mum would be my carer for the rest of my life. It’s better, it’s just better [4]”*.

#### Theme 2: Don’t get me wrong—it was a tough road

All participants described considerable challenges in the post-operative period, including pain, difficulties with dietary adaptions, gastrointestinal side effects and, for two participants, mental state instability.

“I was on antipsychotics and antidepressants. Quite big doses … and then with the general anesthetic I was delusional, hallucinating after the surgery … To start with I was a bit screwed up. A bit angry and found it hard, but I’m so glad I did it [5].”

The negative sequelae anteceded the benefits of surgery, and patients described the post-operative period as difficult.

“The surgery itself—it hurt like heck! It was very tough in the first few weeks [4].”

Some participants reported mismatches between their expectations and the reality of their mental health in this period. The gastrointestinal side effects of the surgery often had social implications, which caused social discomfort. Participants gradually learnt what foods they could not tolerate, adjusting eating habits and portion sizes accordingly, which required long-term commitment. In the medium term, two participants reported an increase in alcohol consumption, which they identified as a “transfer” of addictive tendencies from eating to another outlet.

*“I’m an addictive personality. I did have a period where I drank a lot … But anyway, I’ve stopped drinking completely now [5]”*.

#### Theme 3: Peri-operative support is really important

Participants strongly endorsed the importance of psychosocial support after surgery.

“It was hard, but I had the right kind of support and that made it—not better—but I could get through it [4].”

People valued the support of families, friends, surgical and mental health teams. Support was practical (helping out with ADLs after surgery, financial assistance), social (having company at appointments, support groups), psychological (talking therapies), and psychiatric (monitoring of mental state, medication adjustments as necessary). Participants felt that their mental health care was not well integrated with their perioperative bariatric care.

“…After the op, I really thought there wasn’t a lot of back-up. It would have been good to meet a few people and have this support group because you just have to figure it out yourself [7].”

#### Theme 4: The value of surgery

##### 4(a): It was all worth it—do it!

While acknowledging the post-operative challenges (which were significant as discussed above), seven participants were strongly positive about the overall value personal value of surgery (“miracle,” “the best,” “amazing”). They reflected on the life-changing impacts surgery had for them and family members.

“So, my life turned around … It was the best decision we ever made [6].”

These participants all recommended surgery to others with SMI and obesity.

“I would recommend it to anybody … you still have to look after controlling your eating and all the rest of it, but the benefits have been quite significant [2].”

“…I would not ever change what I did. Not ever … I just would not swap for anything, not even for money. That’s how much it has helped me in my life [6].”

##### Theme 4b: It was not worth it—don’t do it

Despite losing weight, two patients considered surgery was not a net success for them. For one [1], this related to post-operative deterioration in mental state, and for another [7], there were various surgical complications commencing with a bile leak (related to coincidental cholecystectomy) requiring percutaneous drainage, and a small bowel perforation requiring further surgery almost two years after her gastric bypass. While she achieved weight loss and resolution of her diabetes, she felt the complications outweighed the benefits.

The other patient experienced a postoperative relapse in mental state, which she attributed to changes in absorption of antipsychotic medication, and which required inpatient care. After an extended period of poor psychiatric control, she opted to have her laparoscopic band removed. She thought people with SMI were in a difficult bind, based on her experience.

“[If people with SMI have surgery] the drugs won’t work and the person will be even sicker. But if [if they don’t have surgery] they gain too much weight then they’ll get diabetes and die anyway … you’ve got to weigh it up [1].”

#### Theme 5: The health system is discriminatory

Surgery was self-funded, which people found expensive. While bariatric surgery is available through the public health system in New Zealand, no participants could access this funding. They felt people with SMI were being excluded either overtly or covertly and this was discriminatory.

“I think that it’s just wrong to group people and say, ‘you’ve had mental illness, not going to do surgery…’ Because for me, I wouldn’t be here if I didn’t have the surgery … I would really like [these attitudes] to change. It saved my life, it can save someone else’s life [4].”

Participants expressed gratitude to their families for their support and to their surgeon for accepting them for obesity surgery after others had declined them on the basis of their SMI. They felt that their surgeon had seen them as people, whereas the public system viewed them as cases of mental illness.

## Discussion

This is the first qualitative study, to our knowledge, that explores the experiences of people with SMI following bariatric surgery. For most participants, bariatric surgery was successful across multiple domains—in many instances profoundly so—and they recommended bariatric surgery to others with SMI and obesity. Participants reported an increase in their self-esteem and self-confidence, which enabled them to socialize more frequently and lead to improvements in their personal and family relationships.

The post-operative period was viewed as a *tough road*, and participants described the need for additional support at this time—practical, physical, and related to their mental health needs. The fact that six participants had “open laparotomy” surgery may have added to this view. This is not so different from the general population undergoing bariatric surgery (35); Jumbe and colleagues recently conducted a qualitative analysis of patients without mental illness, finding patients experienced unmet psychological needs after surgery, which were not well-recognized by health professionals (36).

Obesity is common amongst people with SMI and a major contributor to premature death in this group. In previous research conducted in the same geographical catchment area as this study (37, 38), 74% of the psychiatric inpatients sampled (*n* = 51) were obese, most with medical comorbidities. They were distressed by their weight, reporting negative feelings about themselves including low personal effectiveness and self-stigmatization. However, none had been referred for bariatric surgery.

The participants in this study viewed the health system as overtly or covertly discriminatory against people with SMI, having experienced barriers to accessing publicly funded bariatric surgery on account of their mental illness.

### Limitations

This study has a small number of participants selected by convenience sampling. As is the nature of qualitative studies, it is hypothesis-generating and cannot provide “proof” of the benefits or risks of bariatric surgery, only an account of personal experiences. Those in in our study were a small sample and not representative of the full spectrum of people with serious mental illness. For example, they (or their families) were able to self-fund surgery, meaning they comprised a comparatively socially advantaged group. Furthermore, while five of the participants had psychotic symptoms, none had a primary schizophrenia diagnosis.

Similarly, the number of open surgeries in this group is not typical. More than half our participants had laparotomies, reflecting local practice. However, the majority of contemporary bariatric interventions are performed laparoscopically. The “open laparotomy” technique may be considered a bias, because it may lead to different post-operative experiences.

The qualitative nature of the study means the data comprises personal accounts, not objectives measures, and as such is subject to recall and other biases. Further research is required incorporating the use of validated measures related to well-being, such as quality of life, self-esteem, addictive, depressive and psychotic symptom scales.

Lastly, it is important to recognize that qualitative researchers cannot completely detach themselves from their own personal and professional preconceptions. As noted by Braun and Clark (33), data are not coded in an epistemological vacuum. The researchers who collected and analyzed the data are all health professionals (with mental health expertise), and both the framing of the interview questions and our interpretation of themes may have been influenced by our medical backgrounds.

## Conclusions

This small sample with a long follow-up showed the substantial benefit of bariatric surgery accrued to this group with severe mental illness and obesity, which extended beyond physical domains. Overall, bariatric surgery was effective for weight loss, it improved quality of life, and our participants recommended it to others with serious mental illness. Participants would have appreciated more multidisciplinary post-operative support targeted to their mental health needs. Their pathway to undergo surgery was difficult, and the reluctance by gatekeepers to allow access to publicly funded surgery was considered discriminatory.

Obesity and metabolic syndrome are common amongst people with SMI, who should be able to access evidence-based interventions in the same way as other members of the community. Health providers and funders need to carefully consider how to remove the overt and covert barriers that add to discrimination and impede people with mental illness accessing effective treatments for their physical health. There is a clear need for obesity surgery trials which include participants with SMI to explore the risks and benefits of bariatric surgery. Surgical and mental health services need to work together to support the physical and psychosocial needs of patients after bariatric surgery.

## Data Availability Statement

The datasets generated for this study are available on request to the corresponding author.

## Ethics Statement

The study received ethical approval from the University of Otago Human Ethics Committee (Health), ref H17/116. The patients/participants provided their written informed consent to participate in this study.

## Author Contributions

SE-P is the principal researcher. SE-P, SR, and MH conceived of the study and designed the protocol and semi-structured interview format. RS provided expert advice on bariatric surgery throughout the study but did not participate in data collection or analysis. SG collected data. SE-P, SR, MH, and AT undertook the thematic analysis. AT assisted with drafting the manuscript. SE-P drafted the first version of this manuscript. All authors provided editorial input and approved the final version of this manuscript.

## Conflict of Interest

The authors declare that the research was conducted in the absence of any commercial or financial relationships that could be construed as a potential conflict of interest.
